# Supplementary material for: Role of Wnt Signaling in Mouse Fetal Skin Wound Healing
Source: Biomedicines. 2022 Jun 28;10(7):1536. doi: 10.3390/biomedicines10071536 (PMC9312897; doi:10.3390/biomedicines10071536)
Supplement: Supplementary file 1 [file biomedicines-10-01536-s001.zip › Supplementary_Files.pdf]

Supplementary Material

Figure Legends

Figure S1. Wnt 1 expression in wound by *in situ* hybridization. Expression of Wnt1 at the mRNA level was not observed from E13 to E17. (**a–c**) Scale bar = 200 μm. (**d–i**) Scale bar = 100 μm.

Figure S2. Wnt 10b expression in wound *in situ* hybridization. Expression was observed in the basal layer of the epidermis around the wound on E13 and in the entire dermis on E15 and E17. However, no useful CT value was obtained by real-time quantitative PCR. (**a–c**) Scale bar = 200 μm. (**d–i**) Scale bar = 100 μm.

Table legends

Table S1. Visual analog scale for scar evaluation.
Table S2. Histological evaluation method (modified Manchester Scar Scale).
