# Supplementary figures and images for: Role of Wnt Signaling in Mouse Fetal Skin Wound Healing
Source: Biomedicines. 2022 Jun 28;10(7):1536. doi: 10.3390/biomedicines10071536 (PMC9312897; doi:10.3390/biomedicines10071536)

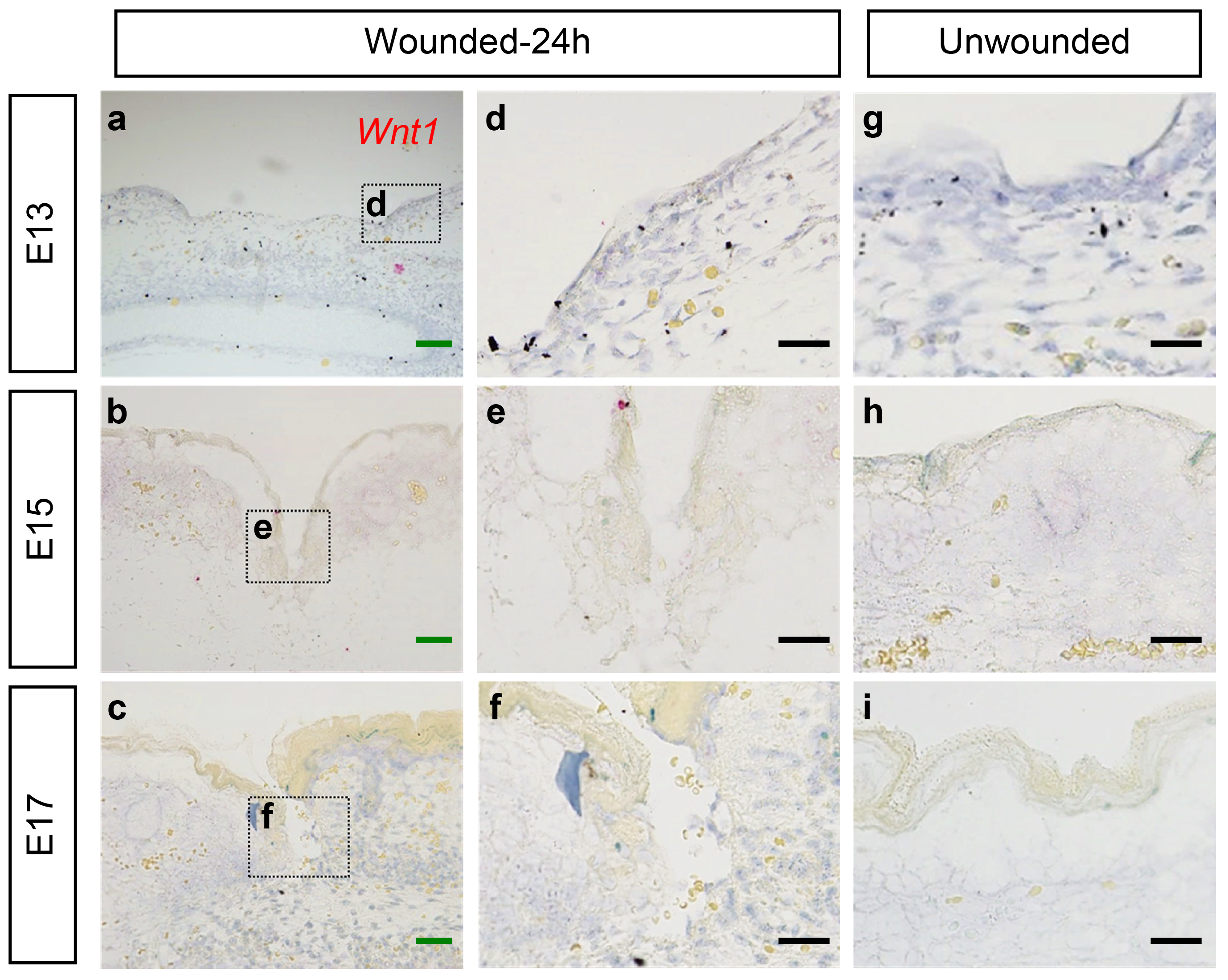

Supplement: Supplementary file 1 [file biomedicines-10-01536-s001.zip › Supp Figure 1.tif]

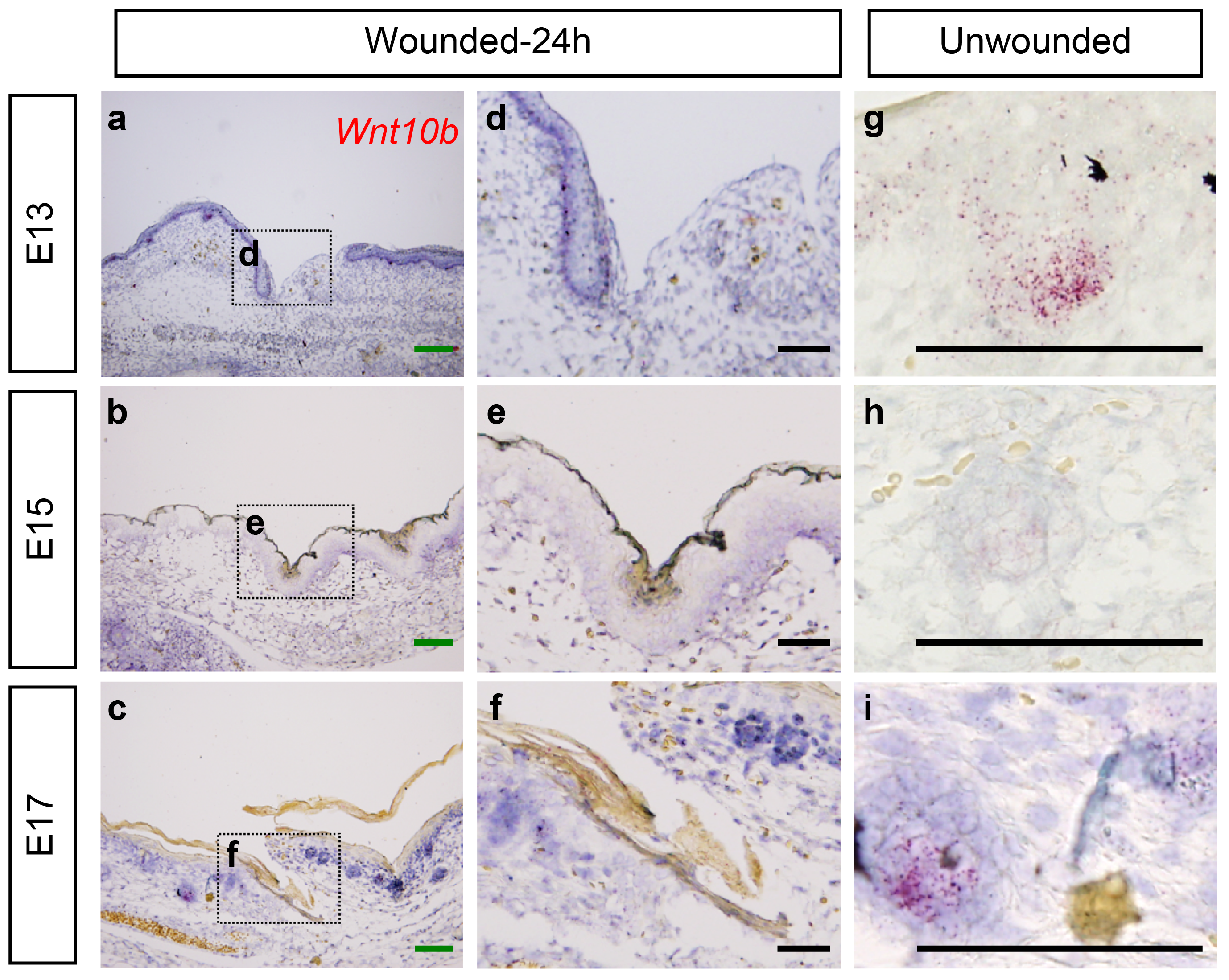

Supplement: Supplementary file 1 [file biomedicines-10-01536-s001.zip › Supp Figure 2.tif]

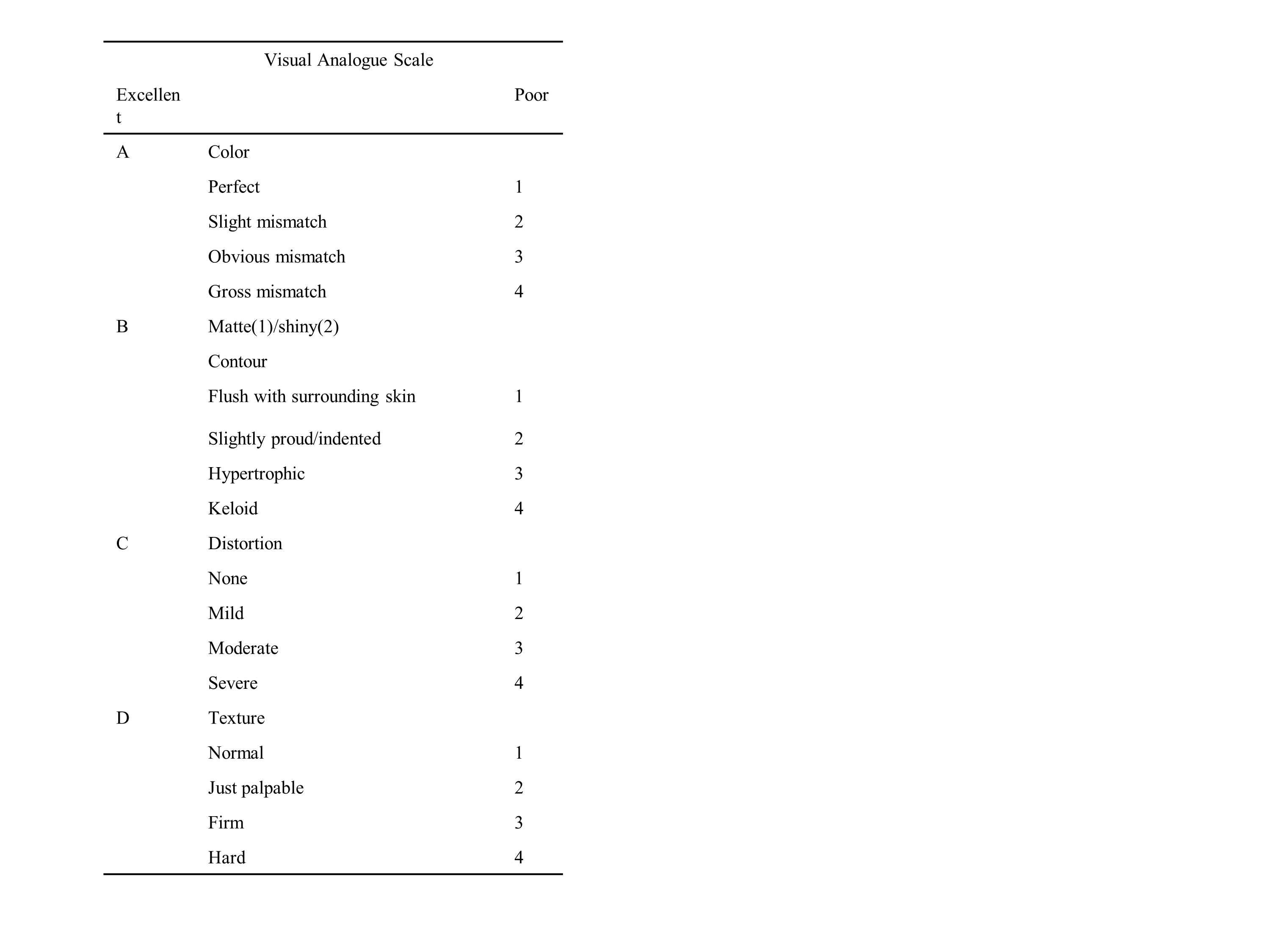

Supplement: Supplementary file 1 [file biomedicines-10-01536-s001.zip › Table S1.tif]

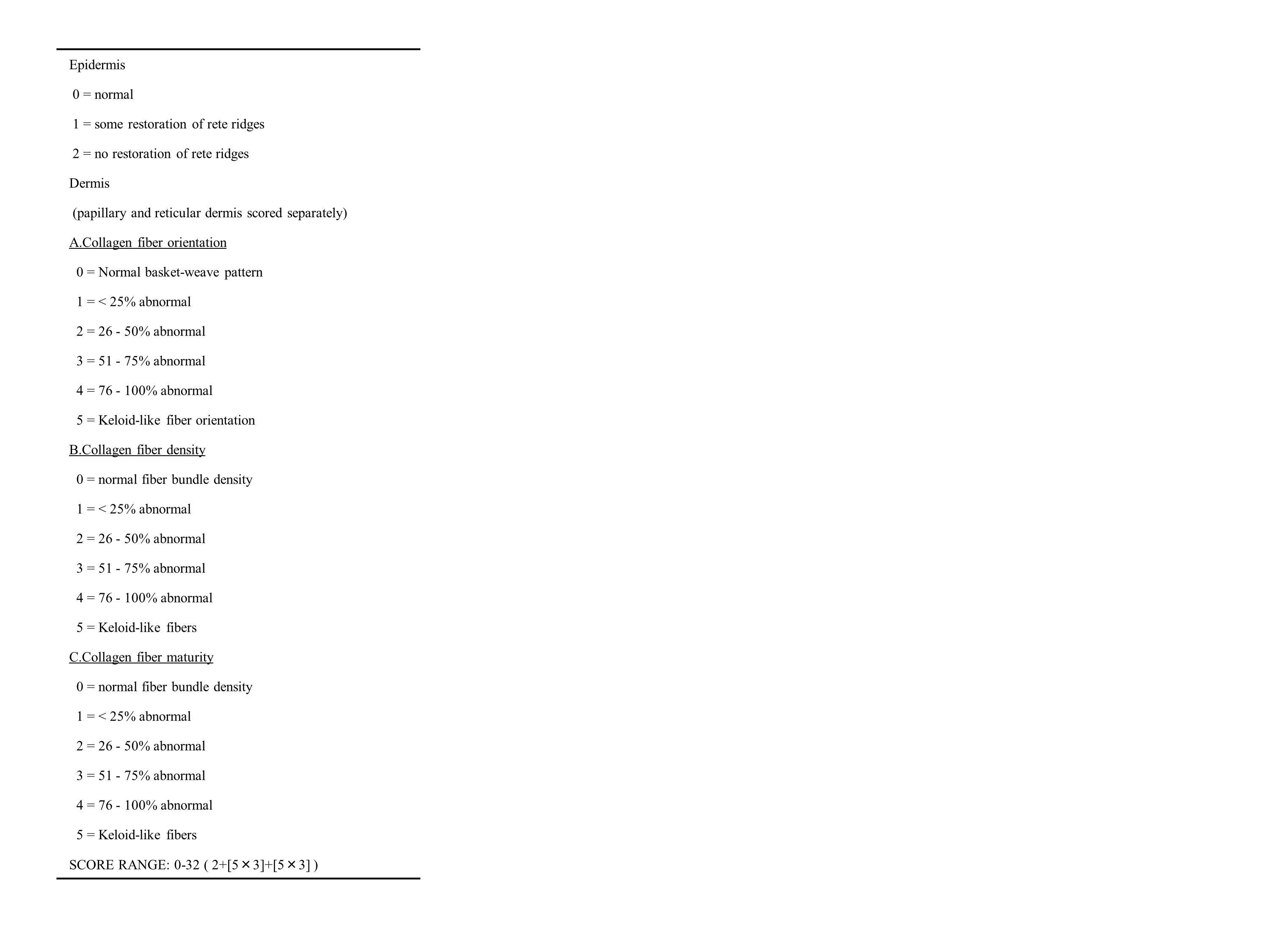

Supplement: Supplementary file 1 [file biomedicines-10-01536-s001.zip › Table S2.tif]
